# Oligopeptide M13 Phage Display in Pathogen Research

**DOI:** 10.3390/v5102531

**Published:** 2013-10-16

**Authors:** Jonas Kügler, Jonas Zantow, Torsten Meyer, Michael Hust

**Affiliations:** Technische Universität Braunschweig, Institut für Biochemie, Biotechnologie und Bioinformatik, Abteilung Biotechnologie, Spielmannstr. 7, 38106 Braunschweig; E-Mails: jonas.kuegler@tu-bs.de (J.K.); j.zantow@tu-bs.de (J.Z.); torsten.meyer@tu-bs.de (T.M.)

**Keywords:** phage display, ORF selection, immunogenic proteins, vaccine, oligopeptide phage display, diagnostic, phage display libraries, genome libraries, antigen discovery

## Abstract

Phage display has become an established, widely used method for selection of peptides, antibodies or alternative scaffolds. The use of phage display for the selection of antigens from genomic or cDNA libraries of pathogens which is an alternative to the classical way of identifying immunogenic proteins is not well-known. In recent years several new applications for oligopeptide phage display in disease related fields have been developed which has led to the identification of various new antigens. These novel identified immunogenic proteins provide new insights into host pathogen interactions and can be used for the development of new diagnostic tests and vaccines. In this review we focus on the M13 oligopeptide phage display system for pathogen research but will also give examples for lambda phage display and for applications in other disease related fields. In addition, a detailed technical work flow for the identification of immunogenic oligopeptides using the pHORF system is given. The described identification of immunogenic proteins of pathogens using oligopeptide phage display can be linked to antibody phage display resulting in a vaccine pipeline.

## 1. M13 Phage Display

**Figure 1:**
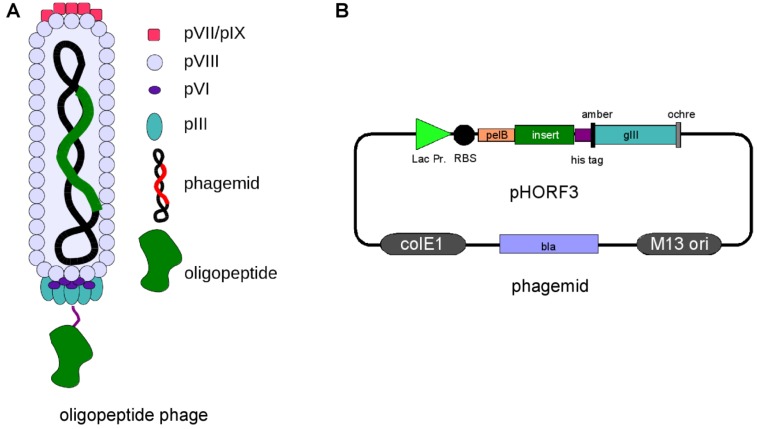
(**a**). Oligopeptide display on phage. pIII=phage protein III. (**b**). Schematic overview of pHORF3. Abbreviations: lacZ promoter: promoter of the bacterial lac operon; RBS: ribosome binding site; pelB: signal peptide sequence of bacterial pectate lyase Erwinia caratovora, mediating secretion into the periplasmic space; gIII: gene coding for the phage protein III; amber: amber stop codon; his: six histidine residues; ochre: ochre stop codon. The elements of the inserts are not drawn to scale.

Phage display technology is a widely used method for the selection of peptides, antibodies and alternative scaffolds [1,2,3,4,5,6], whereas oligopeptide phage display for the identification of immunogenic proteins is, to date, not widely spread. The fundamental technology was invented in 1985 by George P. Smith [7] for filamentous M13 phage. Here, genotype and phenotype of oligo-peptides were linked by fusing the corresponding gene fragments to the minor coat protein III gene of the filamentous bacteriophage M13. The resulting peptide::pIII fusion protein is displayed on the surface of phage particles produced in *E. coli*. The displayed peptides and the corresponding genes can be affinity purified. From the huge diversity of a library, single binding partners are selected under defined selection conditions [8,9,10]. Several phage display systems utilizing insertion of peptide, oligopeptide and antibody genes into the phage genome have been developed, e.g. for phage T7 [11,12,13], phage Lambda [14,15,16,17] and for filamentous phage (f1, fd, M13) [7]. The most commonly phage display systems used for oligopeptide are the bacteriophage lambda [14,18,19,20] and filamentous phage M13 [21,22,23]. A potential disadvantage of the filamentous M13 phage system is the transport of fusion proteins in an unfolded state into the periplasm using the Sec or SRP pathway. In the periplasm the phage coat protein with its fusion partner is folded and incorporated into the phage particle prior to the release of the phage particle from the host cell [24,25]. This can be beneficial for the folding of some proteins, e.g. secreted heterelogous proteins, but also deleterious for other proteins, e.g. cytoplasmic proteins. In contrast, oligopeptides displayed on the lytic phage lambda do not need to be transported to the periplasm and can fold in the cytoplasm but incorporation of large proteins into phage lambda capsids is limited [26,27]. Additionally, the handling and library storage using the lytic lambda phage is laborious compared to the non-lytic M13 phage. Therefore, it is unlikely that a single phage display system will be compatible with all proteins that could theoretically be displayed [28]. A way for displaying cytoplasmic folded proteins using M13 phage is the use of the Tat pathway for secreting the folded protein of interest. In the periplasm, the protein of interest will be coupled to the phage protein III by using a zipper domain [29]. In this review we will focus on the M13 phage display system but will also give examples for lambda phage display in the field of oligopeptide phage display. As described above, G.P. Smith used the phage protein III as fusion partner, but oligopeptides can be fused to all capsid proteins for presentation on the phage surface. The fusion to the coat protein pIII is widely used and allows the display of even large proteins [30]. A peptide library using pIII as fusion partner is also commercially available from NEB (New England Biolabs) [31]. The main coat protein VIII was used for peptides, oligopeptides and antibody fragments [32,33,34,35], but besides other drawbacks the size of the fusion partner is limited [36]. The coat protein pVI was successfully used in some cases as fusion partner for oligopeptides, mainly from cDNA libraries [37,38,39,40,41]. Alternative fusion partners are pVII and pIX, but these fusion partners on the pIII opposing site were mainly used for antibody fragments [36,42,43,44,45,46]. However, the most commonly used fusion partner for different kinds of peptides, oligopeptides and antibody fragments is pIII [36,47]. The genetic information of the gene of interest was first integrated directly into the phage genome fused to the wild-type pIII gene [7,48], but this resulted in a selection drawback of these phage because of lower infectivity of the pIII fusion and a selective growth advantage of phage with smaller genomes. Therefore, display vectors, termed phagemids, have been developed that uncouple peptide expression from phage propagation by providing the gene encoding pIII fusion protein on a separate plasmid, first shown for antibody fragments [49,50,51,52]. The phagemid contains a M13 phage origin of replication for packaging of the vector into phage particle during assembly. The gene of interest replication and expression are uncoupled from the phage replication cycle, leading to a simplification of library amplification. For the production of phage particles a helper phage is needed that provides all additional components for phage replication and packaging. The helper phage genome bears a mutated origin of replication and will be packaged less efficiently into new phage particle [53]. The display of an oligopeptide on phage and the corresponding phagemid (pHORF3) is shown in Figure 1. 

## 2. Panning

**Figure 2:**
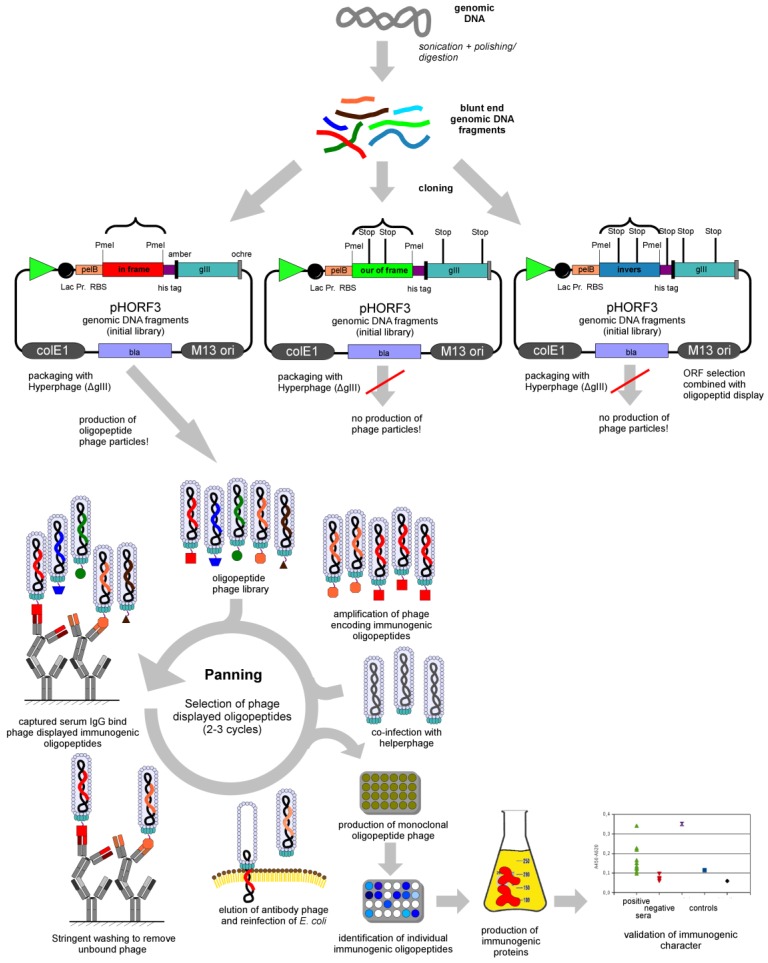
Schematic overview of library construction, ORF selection, panning on captured serum antibodies and validation of the immunogenic character (modified from [23]).

The selection system using phage display was termed panning [54]. For the panning procedure, the desired interaction partner can be immobilised on the surface of microtitre plate wells [23]. An alternative would be the coupling of the targets to magnetic beads [20]. Different types of interaction partners were used to select phage displayed oligopeptides. To identify immunogenic oligopeptides, antibodies of patient/animal sera are captured and used as target for selection [22,55,56,57]. As an alternative commercial polyclonal antibody preparations against pathogens can be used. Bacterial cell lysates [31] were used to select peptides. Also, defined proteins can be used as target, e.g. HIV proteins [58,59]. Afterwards, the phage displayed library will be incubated on the immobilised target. Phage particles which bind weakly and the excess of non-binding peptide phage are removed by stringent washing. Specifically binding peptide/oligeopeptide phage are eluted by trypsin or lowering the pH and used for infection of *E. coli*. For the production of new peptide presenting phage, *E. coli* bearing the phagemid are infected with helper phage. The amplified peptide/oligopeptide phage are used for further panning rounds until a significant enrichment is achieved. Usually two or three panning rounds are necessary to enrich binding oligopeptides. For screening of peptide binders, monoclonal phage are produced for identification in phage ELISA and positive clones are further analyzed by sequencing. Finally, the selected binding partners have to be validated, e.g. the complete protein comprising the selected oligopeptide can be produced and the specificity can be analysed using the appropriate sera [23]. The panning procedure is shown in Figure 2. 

## 3. Genomic Libraries

Oligopeptide phage display libraries generated from fragmented genomes of pathogens allow the expression of a large number of small unknown antigen fragments that can be screened with a convalescent serum-based selection process. For the construction of genomic libraries the DNA is fragmented using ultrasonic sound followed by polishing [22,60], restriction enzymes generating blunt ends [23] or DNaseI digest [18]. The blunt ended DNA fragments are cloned N-terminal of the pIII gene of the phagemid vector. To avoid chimeric DNA fragments, a strategy based on ligation in the presence of a restriction enzyme can be used [61]. The uncut or self-ligated vector is recut but successful ligation of fragments into the vector destroys the recognition site of the restriction enzyme. Dephosphorylation of the DNA fragments prevents cloning of two or more fragments in the same phagemid. For M13 phage display using phagemids, the phagemids containing the DNA fragments are packed into the phage particle and the peptide/oligopeptide::pIII fusion proteins are integrated in the phage coat along with wild type pIII protein provided by the helper phage. Therefore, only a fraction of phage particles present peptide-pIII fusion proteins on their surface. Additionally the cloning of randomly fragmented genomic DNA into phage display vectors requires the in-frame insertion between the signal sequence and the pIII gene of the phagemid vector for expression of the pIII fusion protein. Due to the non-directional cloning, only 1 out of 18 (5.56%) cloned DNA fragments results in an open reading frame (ORF) and additional stop codons in the gene fragments can abrogate the translation of the fusion protein. Therefore, only a minute fraction of phage particle display oligopeptides on their surface and out-of-frame inserts are more efficiently propagated than vectors containing an in-frame insert, leading to an increase in junk clones during selection [62]. To increase the chance of identification of immunogenic oligopeptides using phage display, it is essential to enrich phagemids with in-frame ORFs before panning. Two strategies have been developed for ORF enrichment. The first method uses a fusion to a resistance marker gene. The gene fragments are cloned in front of resistance marker gene to promote the enrichment of gene fragments which are in-frame with the selection marker [21]. The disadvantage of this method is the need to remove the resistance marker gene after ORF enrichment. This second cloning step can lead to a reduced complexity of the library. The removal is done either by sub-cloning of the gene fragment [21] or by flanking the resistance gene with loxP sites which are removed *in vivo* by the Cre recombinase [63]. The second method allows the enrichment of ORFs without any subcloning steps. A helper phage, termed Hyperphage [64,65], with a truncated gIII on the phage genome is used for packaging of the phagemid library. The only source of the pIII coat protein is the pIII-peptide fusion protein encoded on the phagemid and therefore infective phage particle can only be produced if cloned DNA inserts are in frame with the leader sequence and pIII gene [62] (Figure 1). Phage particles produced by co-infection with the Hyperphage display on all pIII proteins the fusion peptide. This polyvalent display allows the enrichment of lower affinity peptides due to avidity effects and the risk of loosing possible binders in the first round of selection, because of over representation of junk clones and phage without peptide/oligopeptide::pIII fusion, is reduced.

## 4. cDNA Libraries

cDNA libraries cover the transcriptome of an organism. The construction of cDNA libraries is hindered by the occurrence of natural stop codons in full length cDNA. Various display systems have been developed to solve this issue. Randomly fragmented cDNA can be used to construct libraries and enrich ORFs as described above for genomic libraries [21]. A C-terminal fusion to the phage coat protein pVI has been described [37]. The phage coat protein pVI is not known to be involved in infection and it is believed that the C-terminus rather than the N-terminus is surface-exposed. In a C-terminal fusion of the cDNA to pVI the presence of stop codons does not prevent the display [37]. A different method to avoid the abrogation of translation is the indirect display on pIII using a c-fos/c-jun attachment [66]. The c-jun leucine zipper is fused to the N-terminus of the pIII coat protein. The cDNA is cloned downstream of the c-fos leucine zipper and the fusion is under control of a separate promoter. The strong interaction between the c-fos/c-jun leucine zippers leads to a linkage of the cDNA translation product and the pIII protein. During assembly of the phage the cDNA - c-fos/c-jun - pIII fusions are incorporated into new phage particle [67].

## 5. Applications of Oligopeptide Phage Display in Pathogen Research

For the identification of immunogenic proteins of pathogens a common method is a 2D-PAGE of cultured pathogens followed by an immunoblot using sera from infected patients or animals which is then analysed by mass spectrometry or microsequencing [68,69,70,71,72]. Weakly expressed antigens or differentially expressed proteins, e.g. dependent on pathogen-host interaction, may not be identified by this method [73]. In these cases, oligopeptide phage display can circumvent these limitations, because oligopeptides can be identified independent of their *in vivo* expression level. Oligopeptide phage display has been used to identify novel immunogenic proteins of different bacteria, e.g. *Mycoplasma* species [22,55,74], viruses, e.g. Cytomegalovirus [19], protozoa [75] and higher eucaryotic organisms [76]. An overview is given in Table 1. 

Beside the identification of immunogenic proteins in pathogen research, oligopeptide phage display is also used in other disease associated fields. Oligopeptide phage display is used for the identification of immunogenic proteins in allergies [56,57,77,78,79]. A related field is the identification of auto antigens in auto immune diseases [39,80]. In cancer research, oligopeptide phage display is used to analyse the humoral immune response on tumor antigens [40,81]. A special application for phage display is the identification of epitopes using oligopeptide phage display. In most cases, peptide phage display libraries displaying 7–15mer random peptides are screened on immobilised antibodies to identify immungenic epitopes. This results only in the identification of short mimotopes (sequences mimicking the epitope) [82,83,84]. Also the identification of conformational epitopes is hampered by the short peptides displayed by random peptide libraries. Here, oligopeptide phage display can be used to identify natural epitopes of pathogens and the chance to detect conformational epitopes is increased by the diverse size of displayed peptides [59,61,85]. A very interesting application of phage display is the identification of *Arabidopsis thaliana* proteins which interact with Pseudomonas species using T7 phage display [13].

**Table 1:** Selected oligopeptide phage display libraries used for pathogen research.

Pathogen/Disease	Target Type	Phage System	Library Type	Reference
*Arabidopsis thaliana* (=host)	*Pseudomonas* cells (=target)	T7	cDNA	[13]
Blue tongue virus (BTV)	two mAbs	M13/ pIII	genomic	[85]
*Cowdria ruminantium*	MAP1 antibodies	M13/ pIII + lambda	genomic	[85]
Human cytomegalovirus (HCMV)	serum	lambda	genomic	[19]
Human immunodeficiency virus (HIV)/ AIDS	GP140 antibody	M13/ pIII	gp140 gene	[59]
*Mycobacterium avium* (MAP)/ Johne's disease (JD)	serum	lambda	genomic	[86]
*Mycobacterium tuberculosis/* tuberculosis	serum	M13/ pIII	genomic	[87]
*Mycoplasma hyopneumoniae/* porcine enzootic pneumonia	serum	M13/ pIII	genomic	[22]
*Mycoplasma mycoides/ CBPP*	serum	M13/ pIII	genomic	[55]
*Mycoplasma mycoides/ CBPP*	serum	M13/ pIII	genomic	[74]
*Mycoplasma pneumoniae/* pneumonia	serum	lambda	genomic	[20]
*Plasmodium falciparum/* malaria	erythrocytes	T7	cDNA	[75]
*Salmonella* Typhimurium/ salmonellosis	serum	M13/ pIII	genomic	[23]
*Streptococcus pneumoniae*/ pneumonia	serum	lambda	genomic	[18]
*Streptococcus pneumoniae/* pneumonia	serum	lambda	genomic	[14]
*Taenia solium/* Neurocysticercosis (NCC)	serum	M13/ pVIII	genomic	[76]

## 6. Technical Work Flow

Here, we give an overview about the work flow to identify immunogenic proteins using the pHORF system which was developed in our group [22,62]. The complete procedure is given in Figure 1. First the genomic DNA of the desired pathogen has to be isolated. If the genomic DNA is limited, the material can be amplified with a commercial genomic DNA amplification kit. The genomic DNA is then used for the digestion with a panel of different blunt-end restriction enzymes or is fragmented by sonication. For both methods, time course experiments have to be made to get DNA fragments of 100–500 bp in size. In general, sonication delivers more random fragments and will better cover the complete genome compared to the use of restriction enzymes. The use of restriction enzymes limits the amount of different gene fragments depending on restriction sites. In addition, many genes will not be in frame with the gIII in the vector resulting in a loss of these gene fragments and an incomplete coverage of the genome. Interestingly, for some genomes sonication leads to very low transformation rates compared to the use of blunt end restriction enzymes. When using sonication, the DNA has to be polished to get blunt ends. Afterwards, the DNA will be ligated into the pHORF3 phagemid vector. The vector has a blunt end restriction site for library cloning. Here, a panel of transformations have to be performed to get a sufficient library size (e.g. 10^6^–10^7^ independent clones). The transformation rates for genomic random fragments is about 10x lower compared the transformation rates for antibody fragments. After transformation, the library will be packaged using Hyperphage [64,65]. The Hyperphage genome is lacking gIII. Hence, the phagemid is the only source for pIII, which is neccassary to assemble infective phage particles. pHORF phagemids which have no genomic DNA insert in-frame with gIII will not be packaged. In theory, only one out of eighteen inserts are in frame with gIII (5' frameshifts, 3' frameshifts, wrong insert orientation). This open reading frame selection improves library quality and facilitates the selection of binding partners from a vast number of non-binding partners. The packacked library will be used for the panning procedure. Here, a purified species specific anti-immunoglobuline antibody will be immobilised in microtitre plates and afterwards the patient/animal serum will be captured. The oligopeptide phage display library will be incubated and then eluted using trypsin. The elution with trypsin is more efficient than the elution with low or high pH. The pHORF vectors encode a trypsin site directly upstream of gIII. Subsequently, the eluted phage particles will be amplified and normally three panning rounds will be performed. After each panning round, the eluted phage particles will be titred. For screening of individual clones, the ELISA set up will be turned around. Colonies from the titre plates will be picked and the monoclonal oligopeptide phage particles will be amplified in microtitre plates. Afterwards, microtitre plates will be coated with anti-pVIII antibodies and the phage production will be captured. A direct coating of the phage production leads to a higher background, especially when the phage production is not PEG purified. When using the anti-pVIII capturing, a purification of the microtitre plate phage production is not necessary. Subsequently, the serum will be added, followed by the species specific horseradish peroxidase conjugated anti immunoglobuline antibody for detection. The serum has to be preincubated with *E. coli* cell lysate and wild type phage, because sera might contain anti- *E. coli and* anti-phage antibodies. To estimate the background of the detection system, controls have to be added. One control should be captured Hyperphage and another control could be a non-relevant oligopeptide phage. Finally, the clones with a good (two-fold to three-fold over background) signal to noise ratio will be sequenced. Now, immunogenic oligopeptides are identified. An important step is the validation of the immunogenic oligopeptides to ensure that no artefacts were selected. Here, a good strategy is the cloning of the corresponding complete protein or protein domain and expression in an adequate production system. The purified proteins can be analysed with positive and negative sera. Depending on the project, the availability of these sera is the critical step. The classification of these sera depends on other diagnostic assays which can be wrong. In addition, for some diseases positive sera are rare or negative sera are not really negative. Finally, the validated immunogenic proteins can be used for further developmental steps.

## 7. Summary

Oligopeptide phage display has proven its power in several disease related fields which led to the identificationof various new antigens. The procedure is complementary to the widely spread classical identification of immunogenic proteins by pathogen cultivation, 2D-PAGE, immunoblot and mass spectrometry. Phage display allows the identification of immunogenic proteins which are low abundant proteins and which are only expressed during pathogen-host interaction. A limitation of oligopeptide phage display is the display itself. Oligopeptides which cannot be displayed on phage cannot be identified. This can be partially circumvented by applying display systems using different signal peptides. A further restriction of a prokaryote based system is the unability to display oligopeptides with posttranslational modifications, e.g. glycosylations. A major advantage of phage display libraries is the enormous number of different oligopeptides presented on phage that can be screened simultaneously. The development of ORF enrichment methods has improved the oligopeptide phage display system considerably making the screening of whole genomes for specific protein protein interactions possible. Phage display systems allow the identification of immunogenic peptides in a short period of time and only a manageable effort is needed. Newly identified immunogenic proteins provide information about the host-pathogen interaction and could be used for the development of new diagnostic tests and vaccines. The combination of oligopeptide phage display with antibody phage display leads to a complete vaccine pipeline. This pipeline can deliver new antigens for diagnostics and therapy as well as tailor made antibodies for diagnostic assays and passive immunisation.
